# Accessing the Influence of Perceived Value on Social Attachment: Developing Country Perspective

**DOI:** 10.3389/fpsyg.2021.760774

**Published:** 2021-10-14

**Authors:** Qingqing Wang, Maosheng Yang, Wensong Zhang

**Affiliations:** School of Economics and Management, Beijing Jiaotong University, Beijing, China

**Keywords:** attachment theory, perceived value, social attachment, sense of belonging, privacy concerns

## Abstract

Perceived value has a positive impact on users’ social attachment in social media usage contexts and is a topic at the forefront of current research in consumer behavior. Although studies have begun to investigate the factors influencing social attachment, there is a lack of research on how perceived value affects social attachment. Therefore, this study uses privacy concern theory, to build a theoretical model with moderated and mediation roles, using Chinese Tik Tok users as data and survey sample, and applying Mplus7.0 to analyze the mediation mechanism and boundary conditions of the relationship between perceived value and social attachment through the structural equation model. In Study 1, data were collected from 600 Tik Tok users to verify the mediating role of the sense of belonging in perceived value and social attachment relationship. The users participating in the questionnaire survey were mainly from mainland China. In Study 2, two waves of data were collected from 500 Tik Tok users to verify the mediating role of the sense of belonging, and support part of the moderating role of privacy concern. However, except that the relationship between information value and social attachment is inhibited by privacy concern, the relationship between entertainment and social value and social attachment is not regulated by privacy concern. This research examines the practical effects of perceived value in the context of social media use, reveals the internal mechanism of the impact of perceived value on social attachment, and provides a reference for the innovative management and commercial practice of social media.

## Introduction

The new wave of information technology revolution has given rise to the flourishing of social media, making social media a sustainable activity in which everyone can participate (Skoric et al., 2016; Hogan and Strasburger, 2018). On one hand, with the improvement of people’s material living standards and the maturity of communication technologies such as the Internet, the number of social media options available to users continues to grow and their voice in the market gradually increases (Christmas, 2021). On the other hand, the rapid increase in market demand and the stimulation of the technological revolution dividend have resulted in more intense competition for social media (Irwin et al., 2021), leading to the emergence of similar social media APPs in the market, which have sprung up in the lives of the public. This means that competition is driving social media platforms to consider how to meet the needs of their users and to increase their value as the starting point of their business in order to obtain sustainable development. Further research shows that spatial distance and physical separation are weakened in the context of social media use, and that users gain a variety of perceived values such as emotional, informational and entertainment value (Wang and Huang, 2017; Gomez-Galan and Martinez-Lopez, 2020). At this point, perceived value serves as a stimulating element that allows users’ needs to be continuously satisfied, thus forming a sustainable usage behavior pattern dominated by social attachment. Clearly, in the face of increasing competition, enhancing the perceived value of users is a key issue in attracting and retaining users to promote the sustainable development of social media. The present study focuses on the following aspects of the relationship between perceived value and social attachment.

Firstly, in the context of social media usage, perceived value refers to the user’s overall preference and comprehensive evaluation of the goods or services involved in a video based on existing subjective impressions when viewing the video on a mobile short video social application (Wang and Huang, 2017). A higher degree of perceived value indicates that users get more pleasure and satisfaction in their interactions with others through self-expression and presentation in the process of using social media, thus establishing a good emotional connection with social media (Hsu and Lin, 2016). It has been argued that perceived value essentially reflects users’ subjective perceptions of the specific value that using social media brings to them (Wang and Huang, 2017; Casalo and Romero, 2019) and plays an important role in the formation of affective attachments (Li and Gao, 2019). Thus, social attachment may be influenced by perceived value, which produces specific satisfaction of various needs of the user, which in turn influences the user’s perception of social media; and treats the self as part of social media, and with the continuous satisfaction of individual perception, the user develops emotional attachment to social media and continuously invests time, energy and even money in social media. This investment ultimately strengthens the bond between the user and the social media, creating a sense of closeness and dependency, which in turn leads to a strong long-term connection (Yang et al., 2021a).

Secondly, previous research suggests that a sense of belonging is a necessary condition and a prerequisite for continued use of social media (Duffett, 2020; Chen et al., 2021; Yang et al., 2021b). So, does the perceived value inspired by users’ use of social media affect their social attachment through the mediating effect of belongingness? Belonging theory suggests that the users’ emotional value gaining self-expression and care from others through social media application platforms will strengthen the emotional connection between users and social media, the essence of which is that users want to be recognized and accepted by social media, making users feel the value of their existence in the virtual society (Hsu and Lin, 2016). This increases users’ reliance on and satisfaction with social media, which in turn enhances their sense of identity and belonging to social media, laying a positive impact on their emotional use of social media. Related studies have also found that users’ perceived value does affect social attachment between users and social media through the mediating effect of belongingness brought about by using social media (Yang et al., 2021b). Thus, the perceived value inspired by social media use may lead to an indirect positive effect on users’ social attachments through the mediating effect of belonging.

In addition, in the context of social media usage, individuals are concerned about who has access to their private information and how this information will be used. The exact attitudes and behaviors of users when confronted with privacy concern also depend on their awareness of privacy protection (Tsai et al., 2017). For example, users will abandon the use of social media once they believe that the scope and extent of their access and the use of personal information exceed their permitted risk tolerance threshold (Tang et al., 2019). As can be seen, users’ privacy concern in social contexts are extremely important, as they help to reduce the likelihood of personal privacy information leakage, reduce the possible negative impact of personal information leakage, and enhance privacy protection, which has become an indispensable moderating variable in big data era. Therefore, based on privacy concern theory, this study proposes that privacy plays a moderating role in the “perceived value-social attachment” relationship (Ayaburi and Treku, 2020).

Despite these valuable results, there are few studies in current literature that have explored and examined the mechanisms and boundary conditions underlying the effects of perceived value on social attachment: First, what effect does perceived value have on social attachment? What are the mediating mechanisms through which this influence is transmitted? Second, does this mediating mechanism show different patterns of action in different management contexts? Third, most previous studies have taken social media in developed western countries as a sample, and there is a lack of research on the factors influencing the perceived value of social media in China. Although these inferences can be drawn from both practical and theoretical perspectives, the deeper theoretical logic remains to be explored, and the pathways through which perceived value works remain to be explored.

To this end, this study constructs a theoretical model of the impact of perceived value on social attachment in the context of the use of social media. The social media Tik Tok is used as the research object, and its mass users are targeted as effective samples. Structural Equation Modeling (SEM) is applied to explore how the different modes of perceived value lay down a driving effect of social attachment, under the mediation of the sense of belonging and the moderation of privacy concern. Through in-depth exploration of its internal theoretical logic and influence mechanism, it will provide practical guidance mechanism formed by social attachment in the social context, and provide theoretical support to the role factors contributing in the formation of users’ social attachment.

## Theoretical Foundation and Hypothesis Development

### Theoretical Foundation on Privacy Concern

With the increasing use of the Internet, social networks and other forms of information sharing, the concern for privacy has gradually become the focus of many studies and discussions. In the existing literature, privacy is defined as “an anxiety about personal privacy” (Yun et al., 2019). Another definition describes it as “concern about controlling the acquisition and subsequent use of personal information” (Tan et al., 2012). Throughout the development of social media, researchers paid special attention to privacy issues in social media (Fogel and Nehmad, 2020). Some researchers tried to evaluate the impact of information disclosure and privacy options of social media on user privacy control. Acquisti et al. (2006) conducted a survey of more than 4,000 Facebook users to study the patterns of information disclosure in online social networks and their impact on privacy. The survey results show that although students’ privacy may be attacked in many ways, only a small percentage of students have changed their privacy preferences. As to the reason of the shortage of social media privacy control, Choon (2018) believe that the privacy options provided by social media do not provide users with the required flexibility to deal with conflicts with friends with different privacy concepts. The study proves that there is often a disconnection between users’ desire to protect privacy and their privacy control behavior. This phenomenon is called privacy paradox (Chung et al., 2021).

Despite the growing interest in research on privacy concern in social media, there is little information and empirical evidence on how privacy concern affects the acceptance of social media (Dienlin and Trepte, 2015). In fact, when users have stronger privacy concern about social media, they are more likely to inhibit the frequency of usage, which undermines the likelihood of continued usage. Therefore, this study aims to explore the impact of social media users’ persistence intention from the perspective of privacy concern, in order to understand the boundary conditions of privacy concern on users’ persistence in using social media, and to provide valuable references for social media developers to design their products and develop marketing strategies, so as to provide more effective services to social media users.

### Hypothesis Development

#### Perceived Value and Social Attachment

Perceived value, a concept based on consumers’ subjective impressions, reflects their evaluation of the perceived benefits and losses of a kind of good or service (Petrulaitiene and Jylha, 2015). However, unlike the graphic information in traditional virtual social environment, the video information in the social media context makes the value perceived by users more intuitive, rich and real, so that users can express their emotional connection with the social media platform in various forms, such as likes, comments and retweets, etc. Therefore, based on the social media usage context, social value, entertainment value and information value are generally used to represent perceived value (Yang et al., 2021b). The perceived value accumulated in this relationship is seen as an extension of the self and has a significant impact on social attachment. As perceived value increases, so does the emotional connection users have to social media (Li and Gao, 2019). This is because the emotional connection created by the interaction between the user and the social media in the context of social media use satisfies the user’s need for perceived value, creating perceptions, memories and a sense of belonging, which leads to a social attachment to the social media (Yang et al., 2021b). Thus, in the context of social media use, perceived value makes users attach to the social media. Specifically, the three perceived values of social value, entertainment value and information value in the context of social media use have an impact on users’ social attachment.

Firstly, social values are mainly the subjective feelings that individuals have about satisfying their self-esteem and enhancing their social identity, reflecting the importance they attach to their self-image and their expectations of social identity (Petrulaitiene and Jylha, 2015). In the context of social media use, when users feel the need to respond positively to positive interactions, they may feel guilty if they fail to respond effectively, and will therefore respond positively through interactions in order to gain approval and get attention from others. This psychological commitment drives users to perceive that they are needed, which in turn leads to social attachment to social media.

Secondly, entertainment value refers to individuals finding interesting contents to spend their free time on and to interact with friends for pleasure (Lee et al., 2014). The study of Jin et al. (2019) finds that entertainment value can effectively influence the occurrence of attachment in intimate relationships and has a specific impact on their behavior in virtual socialization, and that the long-term interactive behavior engaged in between users and virtual communities can satisfy users’ need for emotional pleasure and stimulate positive emotions, fondness and passion, etc., toward that virtual platform. Thus the long-term interactions between users and virtual communities can satisfy users’ need for emotional pleasure, stimulate positive emotions, love and passion, and thus create attachment to the virtual platform (Jin X. L. et al., 2021). It can be seen that in the context of social media use, social attachment is formed by factors such as the user’s entertainment value to be satisfied, and has a specific impact on their individual behavior.

Thirdly, information value indicates the degree of practical convenience that users bring to themselves through their use of social media. Users can find news and other information to keep them informed and to enhance their relevant skills that interest them from social media platforms (Cheung et al., 2014), which in turn generate positive emotions or feelings. Users are able to access more valuable and relevant information in a timely manner, and can share the activities they participate in on the social media platform through functions such as commenting and retweeting, and the level of convenience in accessing the information value (Wang et al., 2017; Jin Y. et al., 2021). The higher the actual level of convenience perceived by users and the greater the information value obtained, the higher the level of attachment of users to social media.

Based on the above reasoning, we hypothesize that:

Hypothesis 1a: Users’ social value is positively related to social attachment.
Hypothesis 1b: Users’ entertainment value is positively related to social attachment.
Hypothesis 1c: Users’ information value is positively related to social attachment.

#### Perceived Value and Sense of Belonging

Research has shown that the perceived value of social media users has a significant effect on their sense of belonging (Tirukkovalluri et al., 2020). Davis (2015) examined the relationship between belongingness and perceived value based on social and behavior theoretical perspectives in a study on the willingness and behavior of sustained engagement on social networking sites. The study was conducted on Facebook and data was collected from 403 Jordanian undergraduate and postgraduate students through a questionnaire, and the extended theory was tested using statistical analysis, which showed a significant effect of perceived value on belongingness (Al-Debei et al., 2013). Al-Debei et al. (2013)’s study further found that a sense of belonging facilitates users’ continued use behavior. Building on Al-Debei et al. (2013)’ s study, Zhang et al. (2017) examined the effects of direct and indirect network externalities on users’ perceived value (including social value, information value, emotional value and hedonic value), sense of belonging and persistence intention based on a survey of WeChat users, using structural equation modeling to analyze the data. The results confirmed the mediating role of belongingness in the influence of perceived value on persistence intentions. It can be seen that users’ perceived value has an influential role in the sense of belonging.

A sense of belonging reflects an individual’s experience of participating in an environment, an experience that makes the individual feel integral to that environment, a feeling that arises from the influence of the external environment on the individual, and the individual’s behavior in the external environment is a result of that influence (An and Liu, 2014; Gao et al., 2017). Belongingness has been extensively studied in both physical and online virtual environments (Zhao et al., 2012). However, unlike traditional virtual community usage contexts, this study focuses on the contexts of social media use and explores the emotional responses of perceived value affecting the emotional bond between users and social media. There are multidimensional content characteristics of users’ perceived value in social media use contexts. In summary, users’ perceived value contains social value, entertainment value and information value, etc., and the higher the degree of perceived value during usage, the stronger the users’ sense of belonging to the social media (Hsu and Lin, 2016; Yang et al., 2021b).

First, social value refers to a subjective evaluation and feeling of individuals toward social media, and reflects not only a certain value attribute inherent in the social media itself, but also a user’s self-perception of the social media (Zhang et al., 2017). In the process of social media use, when the perceived social value is strong, users focus more on learning about other users’ behavior and develop herding behavior, and stronger sense of identification and belongingness to social media use driven by herding behavior (Balakrishnan and Shamim, 2013; Kim and Lee, 2016). Therefore, individuals’ identification with other individuals is enhanced under the effect of herding behavior, which consequently leads to an increase in the degree of belongingness of individuals (Yang et al., 2021b). Therefore, the social value of users has a significant impact on their sense of belonging.

Second, in today’s society, the use of social media has become a priority which enables users to gain physical and mental relaxation and increases the entertainment value of their social situations (Wang and Huang, 2017). Therefore, in the process of human-computer interaction, the individual is also able to experience leisure and entertainment in the real environment, which makes the users comfortable and satisfied as if there is no real-life interpersonal communication and interaction at stake (Jarman et al., 2021). When users are immersed in a social media use context, good entertainment value is generated (Li and Gao, 2019; Yang et al., 2021b). Entertainment value can give users a sense of presence and enhance their entertainment experience during the use of social media. Therefore, users’ entertainment value has a significant impact on their sense of belonging.

Third, the information value not only helps individuals better perceive the presence of others involved in short video topics, but also allows them to obtain or gather more information through the perception of others’ presence (Yin and Li, 2017). Individuals can further master or enrich their self-understanding of the required knowledge and skills through the relevant information, enabling users to make more accurate judgments about the information and knowledge of the short video itself and other situations, which can enhance their sense of belonging to the social media platform (Yang et al., 2021b). According to relevant research in social psychology, individuals choose to trust and accept the influence of others by observing the way people with intimate relationships behave and by choosing to trust and accept others (Walker, 2020). Therefore, users improve their knowledge of social media and the wealth of information it provides through intimate behaviors between users, thereby enhancing their sense of belonging. It can be seen that the information value has a significant impact on users’ sense of belonging.

Based on the above statements, we hypothesize that:

Hypothesis 2a: Users’ social value is positively related to sense of belonging.
Hypothesis 2b: Users’ entertainment value is positively related to sense of belonging.
Hypothesis 2c: Users’ information value is positively related to sense of belonging.

#### Sense of Belonging and Social Attachment

In the study of social media, belongingness can be defined as the engagement with and perception of social media (Chai and Kim, 2012). It reflects the user’s attachment to the social media (Lin et al., 2014) and describes well the psychological states experienced by social media users or their emotional responses to social media during interpersonal interactions (Guo et al., 2016). Research has highlighted the important role of belongingness in the context of social media use (Zhao et al., 2012; Lin et al., 2014). Chai and Kim (2012) found that a sense of belonging positively influenced individuals’ knowledge sharing behaviors on social media, and Lin (2008) found that belongingness predicted loyalty among online social media members, and Chou et al. (2016) found that social media users’ sense of belonging positively influenced knowledge contribution behaviors and citizenship behaviors on virtual platforms, and Lin et al. (2014) found that a sense of belonging was an important indicator of users’ continued use of social media.

Some studies have shown that with the popularity of the Internet and the development of information technology, social media has become a major tool for users to make friends, chat and disseminate information online, and virtual socializing has become a major lifestyle for the public, satisfying individuals’ needs for social interaction (Osatuyi, 2013), with higher controllability for users (Gao et al., 2017), and in a more convenient form. In this context, although the masses are superficially becoming more friendly and more engaged, the spiritual level of interaction and communication between individuals is gradually decreasing and users are becoming emotionally distant from each other. This partly explains why more and more individuals are seeking a sense of belonging in social media and finding new objects of attachment through interpersonal interactions in social media platforms (Duffett, 2020; Yang et al., 2021b). Currently, new social relationships are being regrouped around social media as a result of information technology, reconfiguring new social relationships and focus of life between people. The tendency and trend of weakening the real life of the masses, offline social and interactive relationships are weakened by virtual social and interactive connections, individuals need to communicate with each other and reconstruct their self-identity through social media and in the process forming attachments to social media (Ifinedo, 2016). Users who have formed a strong sense of belonging to social media will feel empty and lonely staying away from social media, and need to express their existence value and emotional relationship through a sense of “belonging” as a ritual. This is why research has found that users with a strong attachment to social media want to be recognized and accepted by the social media, making them become part of the social media platform, which is essentially a sense of belonging to the social media (Hsu and Lin, 2016). Belongingness enhances the social attachment between the social media and the users, allowing them to immerse themselves in this virtual environment without being aware of it, thus creating a social attachment to the social media (Yang et al., 2021b).

The relationship between users’ sense of belonging and social attachment has been examined through data from the perspective of empirical studies. Related studies by foreign scholars point out that users with a higher sense of belonging are more willing to maintain various types of social relationships on social platforms and have a higher level of attachment to social platforms (Lee et al., 2013). Users can interact, communicate and exchange with others through social media platforms, expressing themselves, seeking care, support, recognition and sympathy in virtual social interaction, thus stimulating a sense of belonging to the social media, which in turn has a positive effect on users’ social attachment to the social media (Baumeister and Leary, 1995). Lee et al. (2013)’s study also shows that users’ sense of belonging can, to some extent, indicate their level of emotional response to social platform use, and that this behavior tendency to reflect the level of emotion can lead users to reach an attachment relationship to the social platform. The implication of this is that there is a relationship between an individual’ s sense of belonging and the attachment feelings of social media users. This view has been further tested and supported by domestic scholars. Taking the popular short-video social media in China as the research object, Yang et al. (2021a) explore the relationship between users’ perceived value and their attachment to the social media and find that users’ sense of belonging has a significant positive effect on their attachment, and the stronger the degree of belonging of short-video social media users, the stronger their attachment to the continued use of short-video social media. Therefore, it is known that the user’s sense of belonging in the context of social media use can have an influential effect on social attachment.

Based on the above reasoning, we hypothesize that:

Hypothesis 3: Users’ sense of belonging is positively related to social attachment.

#### Mediating Role of Sense of Belonging

Based on the above analysis of the effect of perceived value on the sense of belonging, it is clear that users’ perceived value is conducive to their sense of belonging. Also, based on the above analysis of the effect of belonging on social attachment, it can be seen that users’ sense of belonging can positively influence their social attachment. Therefore, it can be inferred that users’ sense of belonging plays an important mediating role in the relationship between perceived value and social attachment. It has been found empirically that perceived value positively influences the attachment relationship between users and social media through the mediating role of belongingness, and that the higher the degree of perceived value, the stronger the users’ social attachment (Yang et al., 2021b). Belonging theory suggests that individuals have a strong need of belongingness (Baumeister and Leary, 1995) and therefore users are motivated to engage in interpersonal interactions to satisfy their need to belong in social media contexts. The use of social media creates a convenient, immediate and fast interaction environment for the public, and the entertainment, informational and functional values that users derive from their interactions positively influence their satisfaction and engagement with the social media, which in turn influences and enhances their sense of belonging to the social media. In other words, users satisfy their need for a sense of belonging through perceived value. An individually fulfilling sense of belonging can lead to positive emotions (Baumeister and Leary, 1995), enhance users’ stickiness and attachment to social media (Kim and Lee, 2016), and promote strong social attachment to social media during interactions. Belongingness reflects the psychological state of users in the context of social media use (e.g., joy, emotion and happiness) and the social attachment they develop to the social media. It is evident that belongingness, as an emotional connection and emotional affiliation created in the use of social media, connects the user’s perceived value and social attachment. Further research finds that when users perceive great benefits from social media in the course of using it, its social, entertainment and information values can satisfy their need for belongingness (Mwencha et al., 2014), which in turn enhances and promotes their social attachment (Kim and Han, 2009).

First, social values facilitate the development of social relationships between users during the use of social media, and high levels of social values help to strengthen emotional bonds between users and are predictable in reducing and alleviating individuals’ feelings of loneliness, thereby increasing their sense of belonging and attachment to social media. Clearly, social value is also an important antecedent factor influencing the continued usage of social media by users. On one hand, studies on herding behavior have argued that belonging is a major reason for individual herding, and in social media use contexts, the social value generated by users’ interpersonal interactions through social media platforms under the influence of herding behavior enhances their sense of belonging to social media and has a positive impact on their social and usage behavior (Balakrishnan and Shamim, 2013). On the other hand, the recognition users receive from others on social media, such as likes, positive comments, retweets and video downloads inspires their confidence in continued use and creates social attachment to the social media through a sense of belonging. Thus, perceived value positively influences the attachment relationship between users and social media through the mediation of a sense of belonging. In other words, the sense of belonging plays an important mediating role in the relationship between social values and social attachment.

Second, entertainment value can immerse users in the context of social media use, and acquiring entertainment value in the process reflects a clear willingness to continue using social media, i.e., users have a stronger sense of belonging to social media (Petrulaitiene and Jylha, 2015). As an integral dimension of user perceived value in the context of social media use, entertainment value is the ability of users to enhance their positive emotions, which in turn influences their outward behavior and performance. Therefore, entertainment value helps to improve the users physically and mentally, enhances the emotional bond between the users and the social media, and promotes the formation of a sense of belonging to the social media (Gefen and Straub, 2004). In addition, the immersion that comes with entertainment value helps to increase the engagement and perception of social media by willing users, i.e., it enhances users’ sense of belonging to the social media, which in turn enhances the efficiency and meaningfulness of users’ communication, allowing for a rich feedback and emotional experience, and then a great sense of attachment (Koufaris, 2002). It can be seen that user entertainment value has a significant effect on belongingness, which in turn has a significant effect on social attachment. Thus, belongingness plays a connecting role in the relationship between users’ entertainment value and social attachment. In other words, the sense of belonging mediates the relationship between users’ entertainment value and social attachment.

Third, information value is one of the most basic perceived values that users acquire in the process of using social media (Zhao and Zhou, 2017). In the context of social media use, information value refers to maximizing users’ access to knowledge or information they need at minimal cost (Cheung et al., 2014). On one hand, users’ efficient and timely search for knowledge or information reflects their need to access to information. This efficient need influences users’ positive attitude and behavior intentions, which can promote a strong sense of belonging to the social media. Furthermore, the sense of belonging itself reflects the extent to which users understand the purpose and willingness to use social media (Sit and Birch, 2014). On the other hand, it has been suggested that a sense of belonging is an important factor influencing users’ use of social media, and that users with a strong level of belongingness will enhance their positive evaluation of and trust in social media, thus further contributing to their sense of attachment to social media (Wang and Huang, 2017). And Yen (2012)’s research shows that when users perceive the information value to be more useful, they develop a greater sense of belonging to the social media, increase the reliability of the information source, and in turn strengthen their social attachment by increasing their level of belongingness. Thus, information value positively influences social attachment through the mediating role of belongingness. In other words, belongingness plays an important mediating role in the relationship between information value and social attachment.

Based on the above analysis, it is clear that perceived value has a significant effect on belongingness in the context of social media use, which in turn has a significant effect on social attachment. It can be seen that sense of belonging plays a connecting role in the relationship between users’ perceived value and social attachment. Therefore, sense of belonging can be found to play an important mediating role in the relationship between perceived value and social attachment.

In view of this, the following hypothesis is proposed in this study:

Hypothesis 4a: Users’ sense of belonging plays a mediating role between users’ social value and social attachment.
Hypothesis 4b: Users’ sense of belonging plays a mediating role between users’ entertainment value and social attachment.
Hypothesis 4c: Users’ sense of belonging plays a mediating role between users’ information value and social attachment.

#### Moderating Role of Privacy Concern

Privacy concern is an individual’s awareness and assessment of the risks associated with the invasion of privacy (Tan et al., 2012; Peng et al., 2018; Chung et al., 2021). Cho et al. (2019) state that privacy concern occurs and is stimulated in specific contexts, and that individuals’ privacy concern is most prominent in situations where personal interests are at stake. Specifically, when an individual’s level of privacy concern is high, he or she will worry about the negative consequences of his or her privacy, which causes the individual to restrain his or her behavior, such as inhibiting the individual from uploading work on social media platforms and discussing issues with other users (Schomakers et al., 2019). Conversely, individuals are more willing to express themselves, demonstrate expertise, and share knowledge in virtual environments when their privacy concern is weak. In the study of virtual contexts, privacy concern plays an important role in the behavior of individuals in the context (Li et al., 2019), and empirical research on privacy concern is becoming a focus and topical issue for scholars (Jin X. L. et al., 2021). Although it has been suggested that perceived value, which measures users’ perceived preference and overall evaluation of social media, affects users’ social attachment (Mikulincer and Shaver, 2007; Yang et al., 2021b), social attachment between users and social media can be strong or weak, and only strong perceived values are associated with strong emotional, fond and passionate attachments (Bowlby, 1982). This means that the prediction of attachment maintenance between users and social media is confounded by other factors, and the moderating role of privacy concern draws sufficient attention to this. In particular, with the increasing use of social media for information sharing, the extent to which users are concerned about privacy directly affects the extent to which perceived value is related to attachment to social interaction. Therefore, this study further explores the moderating role of individual differences in privacy concern in the context of social media use, based on an examination of the influence of perceived value on social attachment.

Increased privacy concern negatively moderates the relationship between perceived value and social attachment (Mahmoodi et al., 2018; Punj, 2019). Research has found that the effect of user perceived value on social attachment in social media use contexts is not necessarily negatively moderated by privacy concern (Peters et al., 2015). This may be due to the fact that as users’ perceived value increases, the benefits (i.e., perceived value) they gain in terms of entertainment, social relationship capital, and emotional support are higher than the risks associated with privacy disclosure, thus somewhat attenuating or ignoring the negative effects of users’ privacy concern (Xu et al., 2019), so the impact of perceived value on social attachment is not weakened by privacy concern. The impact of perceived value on social attachment is not reduced by the weakening of privacy concern. In other words, users’ lack of awareness of privacy protection results in their privacy concern not playing a negative moderating role in relationships where perceived value influenced social attachment. Regarding the lack of privacy protection for social media users, relevant studies have provided an explanation, and Kehr et al. (2015) argue that the increase in users’ perceived value drives them to be too positive and optimistic about the benefits brought by using social media, thus interfering with their final judgment, which leads them to underestimate the risk of privacy leakage. Therefore, although users are aware of the privacy leakage problem in social media, they still continue to choose to use the social media based on the positive influence of perceived value (Li et al., 2018), i.e., the influence of users’ perceived value on social attachment fails to be moderated by the negative effect of privacy concern.

However, it has also been suggested that for those individuals who focus on privacy concern, their privacy concern can diminish the role of perceived value in influencing social attachment. For example, Muhammad et al. (2018), in evaluating the impact of social media disclosure and privacy options on users’ privacy control, find that users choose to discontinue sharing personal information on social platforms due to concerns about the potential disclosure of private information, as they are more concerned about the inconvenience of privacy disclosure in their lives, which in turn negatively moderates users’ perceived value. Wang et al. (2020) argued that privacy concerns become more serious as individuals become more concerned and attentive to privacy leaks and a large amount of personal information is exposed to social media platforms. As a result, users with higher level of privacy concerns are worried about the collection, control and use of personal information in the process of using social media, thus creating a perceived loss factor of using that social media. This implies that users with higher levels of privacy concerns expect social media to enhance privacy protection in order to eliminate anxiety and concerns about personal privacy disclosure, thus negatively moderating the relationship between perceived value influencing social attachment. In particular, as the level of users’ privacy concern increases, so does its negative moderating effect on perceived value influencing continued willingness to social media use.

From the above analysis, it is inferred that the negative moderating effect of privacy concern on the relationship between perceived value and social attachment is not significant for users with low levels of privacy concern in the context of social media use. In contrast, users with high levels of privacy concern have a significant negative moderating effect of privacy concern in the relationship between perceived value and social attachment, but do not have the same lack of privacy protection and security awareness as users with low levels of privacy concern. Therefore, the relationship between perceived value and social attachment is found to be negatively moderated by privacy concern, but the moderating effect of privacy concern on the role of perceived value in influencing social attachment has not yet received sufficient attention and discussion.

Based on the above reasoning, we hypothesize that:

Hypothesis 5a: Users’ privacy concern plays a negative moderating role between users’ social value and social attachment.
Hypothesis 5b: Users’ privacy concern plays a negative moderating role between users’ entertainment value and social attachment.
Hypothesis 5c: Users’ privacy concern plays a negative moderating role between users’ information value and social attachment.

### Overview of the Present Research

**FIGURE 1:**
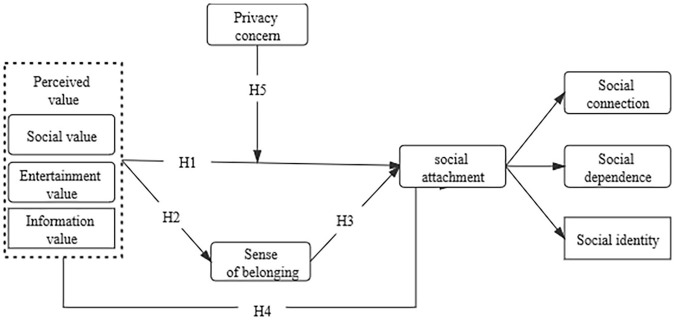
Theoretical model.

To test our theoretical model depicted in Figure 1, we have conducted two studies on user samples using complementary designs to verify our hypotheses. Study 1 uses a sample study of a cross-sectional sample of mainland China and Macau Tik Tok users to verify whether the relationship between perceived value and social attachment can mediate the role of belongingness. Study 2 extends the mediation model of Study 1 by examining privacy concern as a boundary condition, and further verifies the moderating role of privacy concern in relationship between perceived value and social attachment. The model is designed to be tested with samples of Tik Tok users in mainland China collected at two time points. Study 1 tests the mediation model (Hypothesis 1-4), and Study 2 further includes privacy concern to test the entire model (Hypothesis 1-5). Considered together, these two studies comprise a mix of different designs and samples that provide a nice combination of internal and external validity evidence for our theoretical model.

## Study 1

### Method

#### Participants and Procedure

Participants in Study 1 (Tik Tok users) are recruited through https://www.wjx.cn (questionnaire) from mainland China and Macau. The company operates a professional online questionnaire survey platform that focuses on providing users with powerful and user-friendly online designs, a series of services for questionnaires, data collection, and survey results analysis. Compared with traditional survey methods and other survey websites or survey systems, https://www.wjx.cn (questionnaire star) has obvious advantages of custom questionnaire background/logo, red envelope lottery/custom prizes, and automatic data statistical analysis. The validity of using this online questionnaire has been verified in previous studies, and these studies have proved its feasibility and reliability (Jin et al., 2013; Peng and Xie, 2016; Xu and Ma, 2016). Participants access the questionnaire via the hyperlink included in the invitation. Before answering the questionnaire, participants are prompted to carefully read the information sheet, which informed them that participation is voluntary and anonymous, and that only adults living in mainland are eligible to participate. More importantly, this study selected TikTok mass users as the research samples. We chose Tik Tok for the current research for several reasons. First, Tik Tok, as the largest leading social platform in China, has been proved to be feasible and reliable in previous studies. Secondly, Tik Tok is a representative APP, which is a leader in the market segments of social media and possesses the typical characteristics required by the research work. Thirdly, if research findings are supported by the data of TikTok, the current study can just provide management suggestions to improve the commercial practice of social media. Therefore, from this perspective, the use of TikTok public users to conduct data surveys is compatible with our original research purpose.

In order to further ensure that the participants are our target subjects, two filtering questions are used at the beginning of the survey to check the eligibility of the participants (that is, to exclude participants under 18 years old and those with no longer than 6 months of social media use). Since we have pre-screened in the https://www.wjx.cn (questionnaire star) system and only invite adults, no participants are disqualified when the interviewees are double-checked through these two qualification screening questions. Because researchers believe that online questionnaire surveys may cause participants to divorce from practice, we insert three trap questions in the survey as the basis for judging the validity of the questionnaire, which is consistent with the best practices adopted in previous studies (Smith et al., 2016). These trap questions are very simple and have the same format, for example, “Please select ‘2’ for this item”. Only surveys where all trap questions have been answered correctly are considered valid questionnaires. Of the 600 participants, 10.17% (*n* = 61) do not answer the trap question correctly and are therefore excluded from the analysis. Among 539 participants, female accounted for 55.66% and male accounted for 44.34%. The average age is 27 and 23.21% participants were under 20 years old, 38.62% between 21 to 30 years, and 19.52% between 30 to 40 years. Over 41 years old accounted for 18.65%. Unmarried participants accounted for 68.67% and the married accounted for 31.33%.

#### Measures

The measurement items in this study adopt existing mature scales, and carry out translation verification according to the recommendations of the back-translation method, so as to ensure the quality of the questionnaire and its applicability in Chinese context. The measurement items in this study are all measured by Likert 7-point scale. The variables are measured as follows:

##### Social attachment

A 16-item scale, proposed by Yang et al. (2021a), were adopted to measure three dimensions of social attachment, among which 5 items were used to measure social connection, 5 items for measuring social identity and 6 for assessing social dependence. This scale is rated on a 7-point response scale ranging from 1 (strongly disagree) to 7 (strongly agree).

##### Perceived value

The perceived value scale of this study contains three sub-facets and a total of 12 items. Among them, the social value scale refers to related studies by Hagel et al. (1997), including 4 items; the entertainment value scale refers to Overby and Lee (2006); De Vries and Carlson (2014), Kim and Han (2009) including 4 items; the information value mainly refers to the research of Papacharissi and Rubin (2000); Cheung et al. (2014), including 4 items. This scale is rated on a 7-point response scale ranging from 1 (strongly disagree) to 7 (strongly agree).

##### Sense of belonging

The sense of belonging scale of this research mainly refers to the research views of Teo et al. (2003) and Lin (2008), mainly to measure users’ use of social media and sense of belonging, including 4 items. This scale is rated on a 7-point response scale ranging from 1 (strongly disagree) to 7 (strongly agree).

##### Control variables

In the basic personal background information part of the questionnaire, this part is divided into gender, age, marital status, education, occupation, monthly consumption level and usage time according to the characteristics of Tik Tok users, a total of 7 items.

#### Data Analysis Method

In this study, Mplus7.0 is used to do Structural Equation Modeling (SEM) analysis on the collected data. The data analysis includes measurement model analysis and structural model analysis (Bagozzi and Yi, 1988). Specifically, the data analysis methods of this study are described as follows:

First, perform Confirmatory Factor Analysis (CFA). This research uses CFA to test the research model, including item reliability (Item Reliability), composition reliability (Construct Reliability), average variance extraction (Convergence validity), etc., to judge the convergence validity of each aspect. Second, perform discriminant validity analysis. In this study, a more rigorous AVE method is used to test the discriminative validity of the measurement model to verify whether the correlation between the two different aspects is statistically different. Fourth, perform Structural Model Analysis. Including model fit, research hypothesis significance test and explainable variance (*R*^2^) and other results. And on this basis, further analyze the mediating role and moderating role. Third, on the basis of the above analysis, further analyze the mediation effects.

### Results

#### Confirmatory Factor Analysis

Confirmatory Factor Analysis (CFA) is a part of SEM analysis. The variable reduction of CFA measurement model in this study is based on Zhang et al. (2021)’ s two-stage model modification. The measurement model must be tested before performing the structural model evaluation. A complete SEM model report can only be carried out if the measurement model is reasonably acceptable.

In this study, CFA analysis is performed on all dimensions, and the results are shown in Table 1. The standardized factor loadings of all dimensions are between 0.596 and 0.906, and the composite reliability is between 0.794 and 0.912. Convergence Validity is between 0.542 and 0.675, meeting all the standards of Fornell and Lacker (1981) with standardized factor loadings greater than 0.50, Composite Reliability greater than 0.60, and Convergence Validity greater than 0.50 (Verbeke et al., 2014; Hair et al., 2017). Therefore, the model meets the standard, and all aspects have good convergence validity.

**TABLE 1:** Confirmatory factor analysis.

**Construct**	**Item**	**Item Reliability**	**Composite Reliability**	**Convergence Validity**
	
		**STD.**	**SMC**	**CR**	**AVE**
Social value (SV)	SV1	0.711	0.506	0.841	0.572
	SV2	0.664	0.441		
	SV3	0.819	0.671		
	SV4	0.819	0.671		
Entertainment value (EV)	EV1	0.656	0.430	0.862	0.616
	EV2	0.633	0.401		
	EV3	0.901	0.812		
	EV4	0.906	0.821		
Information value (IV)	IV1	0.679	0.461	0.794	0.564
	IV2	0.728	0.530		
	IV3	0.837	0.701		
	IV4	0.616	0.524		
Sense of belonging (SOB)	SOB1	0.615	0.378	0.803	0.580
	SOB2	0.812	0.659		
	SOB3	0.839	0.704		
	SOB4	0.726	0.573		
Social connection (SC)	SC1	0.807	0.651	0.854	0.542
	SC2	0.805	0.648		
	SC3	0.689	0.475		
	SC4	0.631	0.398		
	SC5	0.733	0.537		
Social dependence (SD)	SD1	0.756	0.572	0.886	0.567
	SD2	0.816	0.666		
	SD3	0.704	0.496		
	SD4	0.832	0.692		
	SD5	0.788	0.621		
	SD6	0.596	0.355		
Social identity (SI)	SI1	0.789	0.623	0.912	0.675
	SI2	0.860	0.740		
	SI3	0.805	0.648		
	SI4	0.827	0.684		
	SI5	0.816	0.631		

*Note. STD, Standardized factor loadings; SMC, Square Multiple Correlations; CR, Composite Reliability; AVE, Average Variance Extracted.*

#### Discriminant Validity

The discriminant validity analysis is to examine whether different two variables in the statistics are different or not. In this study, the AVE method is used to evaluate the discriminative validity. Fornell and Lacker (1981) propose the square root of the AVE with the correlation between the construct and other constructs in the model, which means that the variables have discriminative validity. As shown in Table 2, the square roots of the AVE on the diagonal are larger than the correlations between constructs, indicating acceptable discriminant validity. Therefore, study 1 has good discriminative validity.

**TABLE 2:** Discriminant validity for the measurement model.

**Variables**	**Mean**	**SD**	**AVE**	**1**	**2**	**3**	**4**	**5**	**6**	**7**
1.Information value	4.987	0.906	0.564	**0.751**						
2.Social dependence	4.319	1.113	0.567	0.573	**0.753**					
3.Social value	4.739	0.947	0.572	0.647	0.624	**0.756**				
4.Sense of belonging	5.246	0.991	0.580	0.654	0.526	0.592	**0.762**			
5.Entertainment value	5.766	0.863	0.616	0.388	0.312	0.351	0.593	**0.785**		
6.Social connection	4.843	1.139	0.542	0.550	0.441	0.596	0.721	0.434	**0.736**	
7.Social Identity	5.246	0.991	0.675	0.542	0.550	0.441	0.596	0.578	0.593	**0.782**

*Note. The items on the diagonal on bold represent the square roots of the AVE. Off-diagonal elements are the correlation estimates.*

#### Model Fit Degree

In this study, the model fit degree index refers to the model of model fitness analysis, and the 9 most extensive fitness indicators are used for analysis (Jackson et al., 2009). Since the SEM sample is larger than 200, it is easy to cause the chi-square value to be too large and lead to poor fit, so the fit value needs to be corrected by Bootstrap (Bollen and Stine, 1992). After passing the Bollen-Stine Bootstrap correction model, all the fitness indicators in this study have been passed (as shown in Table 3), indicating that the results of this study are acceptable.

**TABLE 3:** Model fit criteria and the test results.

**Model fit**	**Criteria**	**Model fit of research model**	**Result**
χ^2^	The small the better	1101.106	
DF	The large the better	451.000	
Normed Chi-square(χ^2^/DF)	1 < χ^2^/DF < 3	2.441	excellent
RMSEA	< 0.08	0.061	excellent
SRMR	< 0.08	0.052	good
TLI (NNFI)	> 0.9	0.917	excellent
CFI	> 0.9	0.906	excellent
GFI	> 0.9	0.920	excellent
AGFI	> 0.9	0.905	excellent

#### Regression Coefficient

In this research model (as shown in Table 4), social value (SV) (*b* = 0.340, *p* < 0.001), entertainment value (EV) (*b* = 0.085, *p* < 0.001) and information value (IV) (*b* = 0.585, *p* < 0.001) significantly affect the sense of belonging (SOB). Social value (SV) (*b* = 0.663, *p* < 0.001), entertainment value (EV) (*b* = 0.208, *p* < 0.01), information value (IV) (*b* = 0.168, *p* < 0.05), and sense of belonging (SOB) (*b* = 0.612, *p* < 0.01) significantly affected social attachment (SA). Therefore, all H1∼H3 are established.

**TABLE 4:** Regression coefficient.

	**Unstd**	**S.E.**	**Unstd./S.E.**	**Std.**	***p***
*Hypothesis 1*(a)	0.340	0.054	6.314	0.427	***
*Hypothesis 1*(b)	0.085	0.034	2.528	0.138	***
*Hypothesis 1*(c)	0.585	0.062	9.399	0.561	***
*Hypothesis 2*(a)	0.663	0.063	10.552	0.614	***
*Hypothesis 2*(b)	0.208	0.050	4.205	0.226	**
*Hypothesis 2*(c)	0.168	0.047	3.560	0.229	*
*Hypothesis 3*	0.612	0.060	10.231	0.593	***

Note. * *p* < 0.05. ** *p* < 0.01. *** *p* < 0.001.

#### Mediating Effect Analysis

In order to calculate the mediation effect more accurately, this study uses the confidence interval method (Bootstrap Distribution of Effects) to analyze and test the mediation effect. The Bootstrap estimation technique is used to analyze the confidence intervals of the total effect, the indirect effect and the direct effect, and then the significance level of the mediation effect is further calculated (Hayes, 2009). The upper and lower limits of the Bias-corrected 95% confidence interval do not contain “0,” which means the effect is passed. The results of this study show (as shown in Table 5) that the total effect of social value on social attachment is 0.251. At the 95% confidence level, the confidence interval of Bias-corrected is 0.146∼0.386, and the confidence interval of Percentile is 0.138∼0.363, not including 0, so the total effect is established. The indirect effect is 0.069, the confidence interval of Bias-corrected is 0.013∼0.158 at the 95% confidence level, and the confidence interval of Percentile is 0.017∼0.172, which does not contain 0, so the indirect effect is valid. The direct effect is 0.182, the confidence interval of Bias-corrected is 0.061∼0.330 at the 95% confidence level, and the confidence interval of Percentile is 0.031∼0.297, which does not contain 0, so the direct effect is valid. Therefore, it is assumed that H4a holds and is a partial intermediary. In the same way, H4b is established as a partial intermediary. H4c is also established and is a complete intermediary.

**TABLE 5:** The analysis of mediation effect.

**Effect**	**Point Estimate**	**Bootstrap 1000 times**			
		**Bias-corrected 95%**		**Percentile 95%**	
		**Lower bound**	**Upper bound**	**Lower bound**	**Upper bound**
Total effect: SV→SA	0.188	0.212	0.662	0.068	0.428
Indirect effect: SV→SOB→SA	0.206	0.264	0.746	0.038	0.288
Direct effect: SV→SA	0.563	0.152	0.536	0.018	0.527
Total effect: EV→SA	0.167	0.397	0.862	0.170	0.666
Indirect effect: EV→SOB→SA	0.396	0.076	0.492	0.036	0.221
Direct effect: EV→SA	0.205	0.034	0.505	0.261	0.859
Total effect: IV→SA	0.182	0.058	0.415	0.058	0.415
Indirect effect: IV→SOB→SA	0.216	0.095	0.705	0.298	0.806
Direct effect: IV→SA	0.294	−0.091	0.191	−0.052	0.144

*Note. SC, Social connection; SI, Social identity; SD, Social dependence; SV, Social value; EV, Entertainment value; IV, Information value; SOB, Sense of belonging.*

### Discussion

Study 1 provides some evidence for the relationship between perceived value and social attachment. The results of these samples show that when users use social media in the context of social media use, the three dimensions of perceived value (i.e., social value, entertainment value, and information value) have great differences in the effects and mechanisms of social attachment. Specifically, social value can positively affect social attachment, in which the sense of belonging plays a part of the mediating role. Entertainment value can positively affect social attachment, and the sense of belonging plays a part of the mediating role. Information value can positively affect social attachment, in which the sense of belonging plays a completely mediating role. Therefore, when users are in the context of social media use, the sense of belonging plays an extremely important role in the strength of the attachment relationship between the individual and the social media.

Although Study 1 provides preliminary support for the mediating role of belonging between perceived value and social attachment, it has a major limitation in that the key variables are self-reported at the same time, and all variables in the research model are from the same data source. Therefore, the results are affected by the variance of commonly used methods. Although studies have confirmed that its impact is small, it may still increase the reported effect size (Podsakoff et al., 2012). Therefore, in Study 2, we follow the recommendations of empirical sampling research and use the time interval between variables as a method to solve the common method variance problem in empirical research (Ohly et al., 2010; Beal, 2015).

Study 1 verifies the positive effect of sense of belonging on social attachment in the context of social media use. In Study 2, we try to further verify the application of the mediation model in different groups and its boundary conditions. In addition, the more important point is that in Study 2, we include privacy concern to test the entire model, further enriching and expanding existing research fields.

## Study 2

### Method

#### Participants and Procedure

Participants are users of social media in China, and they are mainly recruited through Tik Tok, a short video platform that is popular in the Chinese market. As the largest leading platform in China’s domestic social media industry, this platform has proved its feasibility and reliability in previous studies (Paolacci and Chandler, 2014; Wu et al., 2018; Wang, 2020). At the same time, this study also releases questionnaire recruitment information through WeChat. That is, this study collects data through multi-source surveys. Participants access the questionnaire through a hyperlink. Before answering the questionnaire, they are instructed to read the information sheet. The information tells them that participation is voluntary and anonymous, and only users using Tik Tok for more than 6 months are eligible to participate. In order to ensure that the respondents are our target audience, two filtering questions are used at the beginning of the survey to determine the eligibility of participants (that is, to exclude users under the age of 18 and users who are not continuous user).

In order to reduce common method variance (Podsakoff et al., 2012), we collect data at two time points, where the time interval is 2 weeks. In the first survey, participants report demographic information, perceived value, privacy concern, and sense of belonging. In the second survey conducted two weeks later, participants report the extent of their emotional attachment performance in social situations. In order to stimulate participants’ attention, a monetary reward of $3 is offered, to ensure that the completed questionnaire is returned immediately. We conducted a questionnaire survey on 500 Tik Tok users at time 1, and received questionnaires from 436 respondents (response rate of 87.20%), of which 385 respondents (response rate of 88.30%) responded at time 2 available questionnaires. The average age of these respondents (72.21% women) is 29.25 years (SD = 9.91), and the average use time is 1.32 years (SD = 6.84). Among them, 73.41% is unmarried; 38.44% of the sample has a college diploma (including a high school diploma), followed by a bachelor’s degree (37.66%), a master’s degree (21.04%), and only a few respondents has a doctoral degree (2.86%). According to the independent sample test, among the valid respondents at time 1, those who missed time 2 (*n* = 51) and there is no difference among those who completed the two surveys (*n* = 385) with gender (*t* = −0.32, n.s.), age (*t* = −0.51, n.s.) and usage time (*t* = −0.28, n.s.).

#### Measures

##### Privacy concern

We use Son and Kim (2008); Shin (2010), and Tan et al. (2012)‘ s privacy concern scale, the measurement items for privacy concern was translated from English into Chinese following the back-translation procedure advocated by Cha et al. (2007), and according to the actual use situation of social software, which is modified into a measurement item of privacy, mainly used to evaluate the privacy concern of users in the context of social software use. There are 4 items in total. This scale is rated on a 7-point response scale ranging from 1 (strongly disagree) to 7 (strongly agree).

Perceived value, social attachment, and sense of belonging are measured using the same scales as in Study 1. All measurement items except for demographic questions are responded to on a Likert-type scale ranging from 1 (strongly disagree) to 7 (strongly agree).

##### Control variables

In the basic personal background information part of the questionnaire, this part is divided into gender, age, marital status, education, occupation, monthly consumption level and usage time according to the characteristics of Tik Tok users, a total of 7 items.

#### Data Analysis Method

We have sorted and classified the returned official questionnaires, eliminated invalid questionnaires, and then registered and coded the questionnaires one by one. Specifically, the data analysis methods in this study were as in Study 1.

### Data Analysis and Results

To ensure that the developed questionnaire questions are valid, discriminatory is a very important task in scale development. Therefore, a pre-test is conducted of the data using SPSS 24.0 on the sample collected. The aim is to confirm the semantic fluency of the scale questions, the absence of typos and the appropriateness of the layout. One of the most important tasks is to do an item analysis. Its purpose is to remove topics (or variables) that are not discriminating and use them as a basis for topic improvement.

#### Confirmatory Factor Analysis

As in Study 1, we have performed CFA analysis on all facets, and the results are shown in Table 6. In this study, the evaluation and reduction of CFA measurement model variables are revised based on the two-stage model proposed by Zhang et al. (2021). If the measurement model fit is acceptable, the full SEM model report can be followed.

**TABLE 6:** Confirmatory factor analysis.

**Construct**	**Item**	**Item Reliability**	**Composite Reliability**	**Convergence Validity**
	
		**STD.**	**SMC**	**CR**	**AVE**
Social value (SV)	SV1	0.833	0.694	0.855	0.597
	SV2	0.801	0.642		
	SV3	0.684	0.468		
	SV4	0.765	0.585		
Entertainment value (EV)	EV1	0.772	0.596	0.893	0.676
	EV2	0.793	0.629		
	EV3	0.829	0.687		
	EV4	0.891	0.794		
Information value (IV)	IV1	0.813	0.661	0.903	0.700
	IV2	0.872	0.760		
	IV3	0.847	0.717		
	IV4	0.812	0.659		
Sense of belonging (SOB)	SOB1	0.743	0.552	0.872	0.632
	SOB2	0.875	0.766		
	SOB3	0.846	0.716		
	SOB4	0.703	0.494		
Privacy concern (PC)	PC1	0.709	0.503	0.909	0.716
	PC2	0.881	0.776		
	PC3	0.909	0.826		
	PC4	0.870	0.757		
Social connection (SC)	SC1	0.764	0.584	0.879	0.593
	SC2	0.769	0.591		
	SC3	0.794	0.630		
	SC4	0.857	0.734		
	SC5	0.653	0.426		
Social dependence (SD)	SD1	0.737	0.543	0.895	0.587
	SD2	0.702	0.493		
	SD3	0.855	0.731		
	SD4	0.793	0.629		
	SD5	0.773	0.598		
	SD6	0.726	0.527		
Social identity (SI)	SI1	0.697	0.486	0.887	0.612
	SI2	0.859	0.738		
	SI3	0.871	0.759		
	SI4	0.724	0.524		
	SI5	0.744	0.554		

*Note. STD, Standardized factor loadings; SMC, Square Multiple Correlations; CR, Composite Reliability; AVE, Average Variance Extracted.*

There are eight constructs in this research model, namely entertainment value, information value, privacy concern, social connection, social dependence, social identity, sense of belonging and social value. According to the standards proposed by Anderson and Gerbing (1988), CFA analysis is performed on all constructs. Following the standard proposed by Fornell and Lacker (1981); Verbeke et al. (2014), and Hair et al. (2017), convergent validity is tested with standardized factor loadings greater than 0.50, the composite reliability higher than 0.60, and the average variance extracted higher than 0.50. Therefore, according to the results shown in Table 6, each variable of this study has a good reliability and polymerization validity.

#### Discriminant Validity

Table 7 reports the Discriminant validity for the measurement model, the square roots of the AVE are reproduced on the diagonal. Discriminant validity is the extent to which the measure is not a reflection of some other variables. It is indicated by low correlations between the measure of interest and the measures of other constructs. We have examined discriminant validity using Fornell and Lacker (1981)’s recommendation that the square root of the average variance extracted for each construct should be higher than the correlations between it and all other constructs. Table 7 shows that the squared root of average variance extracted for each construct is greater than the correlations between the constructs and all other constructs. As shown in Table 7, our results support Fornell and Lacker (1981)’ requirement of discriminant validity.

**TABLE 7:** Discriminant validity for the measurement model.

**Variables**	**Mean**	**SD**	**AVE**	**1**	**2**	**3**	**4**	**5**	**6**	**7**	**8**
1.Sense of belonging	3.806	1.161	0.632	**0.795**							
2.Social value	3.835	1.197	0.597	0.531	**0.773**						
3.Entertainment value	4.638	1.207	0.676	0.584	0.595	**0.822**					
4.Information value	4.508	1.276	0.700	0.612	0.454	0.588	**0.782**				
5.Social connection	4.294	1.324	0.593	0.578	0.481	0.597	0.537	**0.770**			
6.Social dependence	3.982	1.268	0.587	0.676	0.576	0.666	0.517	0.623	**0.766**		
7. Social identity	3.640	1.170	0.612	0.620	0.594	0.559	0.538	0.652	0.726	**0.782**	
8.Privacy concern	4.625	1.180	0.716	0.061	0.116	0.077	0.059	0.040	0.047	0.040	**0.846**

*Note. The items on the diagonal on bold represent the square roots of the AVE. Off-diagonal elements are the correlation estimates.*

#### Model Fit Degree

This study reports 9 model fit degree indicators: χ2, DF, Normed Chi-square (χ2/DF), RMSEA, SRMR, TLI (NNFI), CFI, GFI and AGFI, and the results are shown in Table 8. The results show that the model fit degree well, and the research model constructed by the sample data is not significantly different from the actual situation. Therefore, it can be used to interpret the actual observation data.

**TABLE 8:** Model fit criteria and the test results.

**Model fit**	**Criteria**	**Model fit of research model**	**Result**
χ^2^	The small the better	557.735	
DF	The large the better	451.000	
Normed Chi-square(χ^2^/DF)	1 < χ^2^/DF < 3	1.237	excellent
RMSEA	< 0.08	0.025	excellent
SRMR	< 0.08	0.052	good
TLI (NNFI)	> 0.9	0.986	excellent
CFI	> 0.9	0.987	excellent
GFI	> 0.9	0.938	excellent
AGFI	> 0.9	0.925	excellent

#### Regression Coefficient

As shown in Table 9, in Study 2, social value (SV) (*b* = 0.186, *p* < 0.001), entertainment value (EV) (*b* = 0.210, *p* < 0.001) and information value (IV) (*b* = 0.316, *p* < 0.001) significantly affect the sense of belonging (SOB). Social value (SV) (*b* = 0.182, *p* < 0.001), entertainment value (EV) (*b* = 0.259, *p* < 0.001), information value (IV) (*b* = 0.090, *p* < 0.05), and sense of belonging (SOB) (*b* = 0.369, *p* < 0.01) significantly affected social attachment (SA). Therefore, H1∼H3 are established.

**TABLE 9:** Regression coefficient.

	**Unstd**	**S.E.**	**Unstd./S.E.**	**Std.**	***p***
*Hypothesis 1(a)*	0.182	0.043	4.208	0.235	***
*Hypothesis 1(b)*	0.259	0.053	4.867	0.303	***
*Hypothesis 1(c)*	0.090	0.043	2.069	0.114	*
*Hypothesis 2(a)*	0.186	0.051	3.659	0.224	***
*Hypothesis 2(b)*	0.210	0.062	3.395	0.229	***
*Hypothesis 2(c)*	0.316	0.052	6.098	0.376	***
*Hypothesis 3*	0.369	0.060	6.099	0.395	***

Note. * *p* < 0.05. *** *p* < 0.001.

#### Mediating Effect Analysis

We use Bootstrap estimation technology to test the mediation effect according to the recommendations of Hayes (2009), and the research results show (see Table 10) the total effect of social value on social attachment is 0.251. At the 95% confidence level, the confidence interval of Bias-corrected is 0.146∼0.386, and the confidence interval of Percentile is 0.138∼0.363, which does not contain 0, so the total effect is established. The indirect effect is 0.069, the confidence interval of Bias-corrected is 0.013∼0.158 at the 95% confidence level, and the confidence interval of Percentile is 0.017∼0.172, which does not contain 0, so the indirect effect is valid. The direct effect is 0.182, the confidence interval of Bias-corrected is 0.061∼0.330 at the 95% confidence level, and the confidence interval of Percentile is 0.031∼0.297, which does not contain 0, so the direct effect is valid. Therefore, it is assumed that H4a holds and is a partial intermediary. In the same way, H4b is established as a partial intermediary. H4c is also established and is a complete intermediary.

**TABLE 10:** The analysis of mediating effect.

**Effect**	**Point Estimate**	**Bootstrap 1000 times**			
		**Bias-corrected 95%**		**Percentile 95%**	
		**Lower bound**	**Upper bound**	**Lower bound**	**Upper bound**
Total effect: SV→SA	0.251	0.146	0.386	0.138	0,363
Indirect effect: SV→SOB→SA	0.069	0.013	0.158	0.017	0.172
Direct effect: SV→SA	0.182	0.061	0.330	0.031	0.297
Total effect: EV→SA	0.337	0.210	0.464	0.210	0.465
Indirect effect: EV→SOB→SA	0.077	0.018	0.174	0.019	0.176
Direct effect: EV→SA	0.259	0.133	0.386	0.130	0.380
Total effect: IV→SA	0.206	0.096	0.343	0.088	0.337
Indirect effect: IV→SOB→SA	0.117	0.054	0.226	0.049	0.202
Direct effect: IV→SA	0.090	−0.020	0.231	−0.036	0.221

*Note. SC, Social connection; SI, Social identity; SD, Social dependence; SV, Social value; EV, Entertainment value; IV, Information value; SOB, Sense of belonging.*

#### Moderating Effect Analysis

**FIGURE 2:**
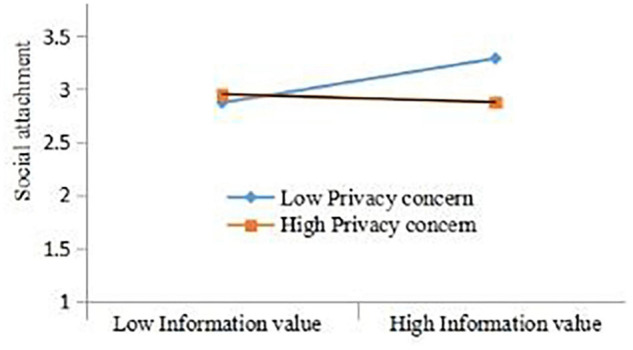
Privacy concern moderating the relationship between social attachment and information value.

In this research model, privacy concern is the moderating variable. The analysis results are shown in Table 11. It can be seen that the moderating effect of SV^∗^PC on SA is 0.017 (*z* = | 0.280| > 1.96, *p* = 0.779 > 0.05), indicating that the moderating effect does not exist. The moderating effect of EV^∗^PC on SA is 0.019 (*z* = | 0.324| > 1.96, *p* = 0.746 > 0.05), indicating that the moderator effect exists. The moderator effect of IV^∗^PC on SA is –0.123 (*z* = | −2.355| > 1.96, *p* = 0.019 < 0.05), which means that the moderator effect exists, which means that for every additional unit of the modulating variable privacy relationship (PC), IV will affect SA The slope of will increase negatively by 0.123 units, that is, negative moderating effect. The result is shown in Figure 2.

**TABLE 11:** The analysis of moderating effect.

**DV**	**IV**	**Estimate**	**S.E.**	***Z*-Value**	***p***
Social attachment	Sense of belonging	0.371	0.088	4.209	***
	Social value	0.192	0.062	3.095	**
	Entertainment value	0.265	0.060	4.442	***
	Information value	0.086	0.062	1.381	**
	Privacy concern	−0.083	0.039	−2.117	*
	Social value × Privacy concern	0.017	0.060	0.280	0.779
	Entertainment value × Privacy concern	0.019	0.058	0.324	0.746
	Information value × Privacy concern	−0.123	0.052	−2.355	*

Note. * *p* < 0.05. ** *p* < 0.01. *** *p* < 0.001.

### Discussion

With the collected data from multi-source survey, results of Study 2 demonstrate that perceived value produces a positive impact on user’s social attachment through sense of belonging. This effect depends on the user’s privacy security, which verifies and expands the results of Study 1. It is worth noting that, unlike Study 1, privacy concern in Study 2 does not all mitigate the overall direct relationship between perceived value and social attachment. There are some potential reasons for this result.

Firstly, compared with Study 1, the further evidence in Study 2 shows that information value can positively affect social attachment, in which the sense of belonging plays a completely mediating role, and privacy concern plays a negative moderating role. Therefore, the user’s sense of belonging to social media becomes more intense, driven by the user’s perceived value and evaluation of social media, which triggers the generation of social attachment. Meanwhile, when an individual, in case of social media use scenarios, has a higher level of privacy concern, the relationship between information value and social attachment is weaker. The higher the level of privacy concern, the weaker the user’s attachment to social media, and the lower the impact of perceived value on social attachment. Conversely, once users do not pay attention to privacy concern, the more various beneficial values and benefits they perceive for social media, then the stronger the user’s attachment to social media. Therefore, as expected, privacy concern acts as a buffering condition and plays an indirect relationship between the information value and social attachment guided by positive emotions.

Secondly, compared to Study 1, further evidence from Study 2 suggests that social value and entertainment value are able to positively influence social attachment, with self of belonging playing a mediating role, but privacy concern does not play a regulatory role. One potential reason why privacy concern has not played a moderating role may be some of its limitations. Privacy in the context of social media use itself does not provide users with effective tools to counter social value and entertainment value. When a person strengthens social media privacy protection measures, and realizes that he or she does not change the perceived value degree, as well as his or her behavioral tendencies and emotional relationship toward social media, although time is consumed, he or she may perceive the benefits brought by social value and entertainment value, this dilutes the reconstruction and protection functions provided by privacy concern. Since preventing these perceived values and their impact on social attachment in the context of social media use may require more powerful solutions, it may be difficult for privacy concern in Study 2 to show buffering.

## Research Results and Discussion

### Theoretical Contributions

Compared with the existing literature, this article is innovative in the following aspects:

Firstly, this article combines perceived value with social attachment, which is an interdisciplinary research. The research on perceived value is currently mainly concentrated in the field of marketing, while social attachment belongs to the field of psychology. Although at the two-way level of theory and practice, the impact of perceived value on social attachment has begun to appear, the theoretical system is still not perfect, and the specific impact mechanism is not fully verified, and it is mostly concentrated in their respective fields. This research breaks the gap of previous related research perspectives, and for the first time clearly focuses on perceived value and social attachment, verifies the impact of perceived value on social attachment, and explores the underlying influence mechanism to inspire further research.

Secondly, this paper explores and examines the boundary conditions of privacy concern while affecting social attachment, under perceived value atmosphere. It is the first time privacy concern as moderator is introduced in social media use scenarios, expanding the existing research on perceived value impact on social attachment and its moderating mechanism, which confirms that there is weakness in the relationship establishment between the users and social media. This study finds that although users are aware that privacy in social contexts may be attacked in many ways, they will not change their privacy preferences and behaviors, in addition to the suppression of the information value on social attachment. This indicates that there is a disconnection between their desire to protect privacy and their privacy control behavior; this phenomenon is called the privacy paradox (Chung et al., 2021). This study enriches the boundary conditions of the effect of perceived value on social attachment.

Thirdly, this article takes social value, entertainment value and information value as three kinds of perceived value for research, which enriches the related research of value theory. Although existing studies have also paid attention to the issue on the unclear relationship between different dimensions and construction of perceived value, and believed that different dimensions can be studied as separate perceived value, they have mainly focused on the impact of perceived value on continued willingness. Research on the attachment relationship between users and social media is still scarce. And this research expands the boundaries of existing research on perceived value theory by exploring the relationship between “how value affects users’ continued use behavior,” and enables new development and application in the context of social media use.

### Practical Implications

This research provides a valuable reference for enhancing users’ perceived value and users’ social attachment establishment, as described below.

Firstly, social media can stimulate social attachment by enhancing users’ perceived value. For social media developers, in order to maintain and improve the perceived value of users, social media can consider implementing relevant management practices. Upgrade and improve short video platform functions, innovate and make breakthroughs in short video content marketing, convert content value and IP value into brand value, identify young people’s consumption desires and consumption ideas, and meet user entertainment needs (such as comedy/jokes). In order to kill time, people enjoy relaxing entertainment in the video swiping process, or get recreational services, which enhances the user’s entertainment value sensing capability and meets the needs of people’s social lives (key opinion leaders and hot content creators). In order to gain recognition and to maintain social relationships, people tend to get information from social talks, which enhances their social value sensing ability and meets their information needs (such as dry goods sharing, celebrities’ adverting of goods and 7-day hottest dynamic product information). In order to be useful, people tend to gain trustworthy and reliable information while swiping short video, which enhances their information value sensing capability. Therefore, relying on the differences in content creation ecology, fan preferences and typical celebrities to match the different needs and motivations of different users, social media can stimulate social attachment by enhancing users’ perceived value.

Secondly, social media can indirectly enhance social attachment by establishing a high-quality sense of belonging. This study shows that users’ perceived value makes use of the mediating role of sense of belonging, and then produces a positive direct impact on social attachment. Just like Maslow’s point of view in “Needs Hierarchy Theory,” “needs of belonging and love” are important psychological needs for people. Only by satisfying “needs of belonging and love” can people realize “self-value.” Therefore, social media platforms can relieve users’ pressure and loneliness through the creation of high-quality content to generate the recognition for social media, to get warmth, help and love from it, thereby eliminating or reducing loneliness, and gaining a sense of security. On the social media platform, by knowing more friends through the platform, users can maintain communication and contacts, and even gain friendship, support and respect, thereby gaining identity and attachment to social media.

Thirdly, when social media platforms strive to improve social attachment by developing perceived value, special attention should be paid to the differences in users’ cultural values (privacy concern). This study shows that when the level of privacy concern is high, users’ privacy concern plays a negative moderating role between users’ information value and social attachment. This reminds social media platforms of attaching great importance to the construction of privacy, security and control in the construction of user perceived value. Launching new functions for privacy issues, such as setting the privacy concern level function, automatically reporting the privacy risks to users, reducing the sense of exposure and intrusion of users’ information, can effectively reduce the main source of privacy concern. Strengthening users’ privacy control awareness, promoting the mode of information disclosure in social networks (for example, unpopular links, links provided in social media platforms showing some information about a certain individual who does not intend to disclose this information) and focusing on the impact of privacy leakage strengthen users’ trust in social media, weaken the negative impact of privacy risks, and build a good social trust.

### Conclusion

Previous studies have shown that perceived value has a positive effect on users’ social attachment in social media usage contexts (Wang et al., 2017; Li et al., 2019). To develop and enrich existing research fields, we have analyzed the driving effect of perceived value on social attachment in the scenarios of social media use, dug how and when users’ perceived value affects users’ social attachment in social context.

Based on privacy concern theory, this research constructs a new theoretical model, uses structural equation model to explore the relationship between perceived value and social attachment, introduces privacy concern to explain the specific impact mechanism, and at the same time pays attention the role that perceived value plays in it. We believe that the perceived value generates a positive impact on user’s social attachment through sense of belonging, and this impact depends on the user’s privacy concern.

There are two research components in this study, and Mplus7.0 is used for measurement model and structural model analysis, as follows:

Study 1 is a questionnaire survey on 600 Tik Tok users from mainland China and Macau, using Mplus7.0 for data analysis. The results support the mediating role of the sense of belonging, and find that perceived value has an indirect positive effect on social attachment through the mediating role of the sense of belonging. Study 2, apart from conducting the two-wave data survey on 500 Tik Tok users to confirm the mediating role of sense of belonging, also verifies the moderating role of privacy concern. However, the moderating role of privacy concern between perceived value and social attachment has not been fully supported. Specifically, except that the relationship between information value and social attachment is inhibited by privacy concern, the relationship between entertainment value and social value with social attachment is not regulated by privacy concern.

### Research Limitations and Future Research Directions

As with any other research, the findings of our study should be interpreted with certain limitations in mind.

On one hand, this study treats social attachment as a general holistic construction to test its influence mechanism. Although it is a common practice in the literature to reduce the sub-facets of the second-order facets through the weighted average method in the analysis, this may cause deviations in the analysis results. This kind of conclusion, although it could reveal the positive interest of perceived value on social attachment in the social context, is difficult to reveal the differences of the different dimensions in social attachment. In consequence, future research can start from the perspective of user perceived value, especially distinguishing the different dimensions of social attachment, and examine the influence of perceived value on social connection, social attachment and social identity and the mediating role of sense of belonging in these relationships.

On the other hand, the questionnaire data of this study comes from a single questionnaire survey website platform, which may threaten the external validity of the research results. “Questionnaire Star” is one of the most popular survey websites, it provides functions equivalent to Amazon Mechanical Turk (MTurk). Although our careful choice of academic research study site “Wenjuan Xing/Questionnaire Star” (a very professional survey questionnaire site in China) alleviates this worry, due to the new virus Covid-19 pandemic caused by the crown restrictions, we cannot confirm whether the results of the current study is universal under other contexts. Therefore, we urge future researchers to replicate this research in other contexts to conduct cross-crowd testing, conduct multi-group comparative analysis, verify the promotion of the research results, and provide appropriate marketing strategies for different sub-groups.

Thirdly, although this study provides preliminary support for the mediation hypothesis, the key variables in this study are self-reported at the same time. Therefore, the hypothesis results of the study are affected by the variance of commonly used methods. Although the effect is statistically confirmed to be small, it is still possible to increase the reported effect size (Ohly et al., 2010). In future research, we propose to address these limitations by using dual-wave online panel samples. In addition, we should also note that for social attachment such psychological phenomenon that contains a strong individual experiences and feelings, to only make quantify research from the horizontal viewing and the relatively static angle is clearly not enough, not all the particular dynamic belonging to social attachment can be perceived. We encourage researchers to adopt research methods with dynamic advantages, such as qualitative research, to better capture and describe the contextual process of social attachment development in detail.

## Data Availability Statement

The original contributions presented in the study are included in the article/Supplementary Material, further inquiries can be directed to the corresponding author/s.

## Author Contributions

MY and WZ involved in conceptualization and involved in writing the review and editing and performed formal analysis. MY and QW involved in data curation. WZ and QW performed investigation. MY, WZ, and QW involved in writing the original draft. All authors have read and agreed to the published version of the manuscript. All authors contributed to the article and approved the submitted version.

## Conflict of Interest

The authors declare that the research was conducted in the absence of any commercial or financial relationships that could be construed as a potential conflict of interest.

## Publisher’s Note

All claims expressed in this article are solely those of the authors and do not necessarily represent those of their affiliated organizations, or those of the publisher, the editors and the reviewers. Any product that may be evaluated in this article, or claim that may be made by its manufacturer, is not guaranteed or endorsed by the publisher.

## References

[B1] Acquisti A. Brandimarte L. Loewenstein G. (2006). “Imagined communities: awareness, information sharing, and privacy on the Facebook,” in *Lecture Notes in Computer Science*, eds Danezis G. Golle P. (Heidelberg: Springer), 36–58. 10.1002/jcpy.1191

[B2] Al-Debei M. M. Al-Lozi E. Papazafeiropoulou A. (2013). Why people keep coming back to Facebook: explaining and predicting continuance participation from an extended theory of planned behavior perspective. *Dec. Support Syst.* 55 43–54. 10.1016/j.dss.2012.12.032

[B3] An Z. Liu L. (2014). The influence factors of SNS users’ sense of belonging: theoretical model and empirical test -a cross-culture study on SNS. *eLife Sci.* 3 332–337. 10.7554/eLife.03726 25310239 PMC4194450

[B4] Anderson J. C. Gerbing D. W. (1988). Structural equation modeling in practice: a review and recommended two-step approach. *Psychol. Bull.* 103 411–423. 10.1037/0033-2909.103.3.411

[B5] Ayaburi E. W. Treku D. N. (2020). Effect of penitence on social media trust and privacy concerns: the case of Facebook. *Int. J. Inf. Manage.* 50 171–181. 10.1016/j.ijinfomgt.2019.05.014

[B6] Bagozzi R. P. Yi Y. (1988). On the evaluation for structural equation models. *J. Acad. Market. Sci.* 14 33–46. 10.1007/BF02723327

[B7] Balakrishnan V. Shamim A. (2013). Malaysian Facebookers: motives and addictive behaviours unraveled. *Comput. Hum. Behav.* 29 1342–1349. 10.1016/j.chb.2013.01.010

[B8] Baumeister R. F. Leary M. R. (1995). The need to belong: desire for interpersonal attachments as a fundamental human motivation. *Psychol. Bull.* 117 497–529. 10.1037/0033-2909.117.3.497 7777651

[B9] Beal D. J. (2015). ESM 2.0: state of the art and future potential of experience sampling methods in organizational research. *Annu. Rev. Organ. Psychol. Organ. Behav.* 2 383–407. 10.1146/annurev-orgpsych-032414-111335

[B10] Bollen K. A. Stine R. A. (1992). Bootstrapping goodness-of-fit measure in structural equation models. *Sociol. Methods Res.* 21 205–229. 10.1177/0049124192021002004

[B11] Bowlby J. (1982). Attachment and loss: retrospect and prospect. *Am. J. Orthopsychiatry* 52 664–678. 7148988 10.1111/j.1939-0025.1982.tb01456.x

[B12] Casalo L. V. Romero J. (2019). Social media promotions and travelers’ value-creating behaviors: the role of perceived support. *Int. J. Contemp. Hosp. Manage.* 31 633–650. 10.1108/IJCHM-09-2017-0555

[B13] Cha E. S. Kim K. H. Erlen J. A. (2007). Translation of scales in cross-cultural research: issues and techniques. *J. Adv. Nurs.* 58 386–395. 17442038 10.1111/j.1365-2648.2007.04242.x

[B14] Chai S. Kim M. (2012). A socio-technical approach to knowledge contribution behavior: an empirical investigation of social networking sites users. *Int. J. Inf. Manage.* 32 118–126. 10.1016/j.ijinfomgt.2011.07.004

[B15] Chen Y. P. Zhu Y. Jiang Y. X. (2021). Effects of admiration of others on social media fatigue: loneliness and anxiety as mediators. *Soc. Behav. Pers.* 49:e10058. 10.2224/sbp.10058

[B16] Cheung C. M. K. Lee M. K. O. Lee Z. W. Y. (2014). Understanding the continuance intention of knowledge sharing in online communities of practice through the post-knowledge-sharing evaluation processes. *J. Assoc. Inf. Sci. Technol.* 64 1357–1374. 10.1002/asi.22854

[B17] Cho H. C. Roh S. Park B. (2019). Of promoting networking and protecting privacy: effects of defaults and regulatory focus on social media users’ preference settings. *Comput. Hum. Behav.* 101 1–13. 10.1016/j.chb.2019.07.001

[B18] Choon M. J. K. (2018). Revisiting the privacy paradox on social media: an analysis of privacy practices associated with facebook and twitter. *Can J. Commun.* 43 339–358. 10.22230/cjc.2018v43n2a3267

[B19] Chou E. Y. Lin C. Y. Huang H. C. (2016). Fairness and devotion go far: integrating online justice and value co-creation in virtual communities. *Int. J. Inf. Manage.* 36 60–72. 10.1016/j.ijinfomgt.2015.09.009

[B20] Christmas C. M. (2021). Concepts of normativity shape youth identity and impact resilience: a critical analysis. *Int. J. Mental Health Addict.* 19 119–133. 10.1007/s11469-019-00141-x

[B21] Chung K. C. Chen C. H. Tsai H. H. Chuang Y. H. (2021). Social media privacy management strategies: a SEM analysis of user privacy behaviors. *Comput. Commun.* 1174 122–130. 10.1016/j.comcom.2021.04.012

[B22] Davis K. (2015). Teachers’ perceptions of Twitter for professional development. *Disabil. Rehabil..* 37 1551–1558. 10.3109/09638288.2015.1052576 26030199

[B23] De Vries N. Carlson J. (2014). Examining the drivers and brand performance implications of customer engagement with brands in the social media environment. *J. Brand Manage.* 21 495–515. 10.1057/bm.2014.18

[B24] Dienlin T. Trepte S. (2015). Is the privacy paradox a relic of the past? An indepth analysis of privacy attitudes and privacy behaviors. *Eur. J. Soc. Psychol.* 45 285–297. 10.1002/ejsp.2049

[B25] Duffett R. (2020). The YouTube marketing communication effect on cognitive, affective and behavioural attitudes among generation Z consumers. *Sustainability* 12:5075. 10.3390/su12125075

[B26] Fogel J. Nehmad E. (2020). Secrets and likes: the drive for privacy and the difficulty of achieving it in the digital age. *J. Consum. Psychol.* 30 736–758. 10.1016/j.chb.2008.08.006

[B27] Fornell C. R. Lacker D. F. (1981). Structural equation models with unobservable variables and measurement error. *J. Market. Res.* 18 382–388. 10.2307/3150980

[B28] Gao W. Liu Z. P. Li J. Y. (2017). How does social presence influence SNS addiction? A belongingness theory perspective. *Comput. Hum. Behav.* 77 347–355. 10.1016/j.chb.2017.09.002

[B29] Gefen D. Straub D. W. (2004). Consumer trust in B2C e-commerce and the importance of social presence: experiments in e-products and e-services. *Omege Int. J. Manage. Sci.* 32 407–424. 10.1016/j.omega.2004.01.006

[B30] Gomez-Galan J. Martinez-Lopez J. A. (2020). Social networks consumption and addiction in college students during the COVID-19 pandemic: educational approach to responsible use. *Sustainability* 12:7737. 10.3390/su12187737

[B31] Guo J. Liu Z. Liu Y. (2016). Key success factors for the launch of government social media platform: identifying the formation mechanism of continuance intention. *Comput. Hum. Behav.* 55 750–763. 10.1016/j.chb.2015.10.004

[B32] Hagel J. Iii J. H. Armstrong A. Hagel Iii J. Armstrong A. G. Armstrong A. H. (1997). *Net Gain: Expanding Markets Through Virtual Communities.* Boston, MA: Harvard Business School Press.

[B33] Hair J. F. Hult G. T. M. Ringle C. M. Sarstedt M. Thiele K. O. (2017). Mirror, mirror on the wall: a comparative evaluation of composite-based structural equation modeling methods. *J. Acad. Market. Sci.* 45 616–632. 10.1007/s11747-017-0517-x

[B34] Hayes A. F. (2009). Beyond Baron and Kenny: statistical mediation analysis in the new millennium. *Commun. Monogr.* 76 408–420. 10.1080/03637750903310360

[B35] Hogan M. Strasburger V. C. (2018). Social media and new technology: a prime. *Clin. Pediatr.* 57 1204–1215. 10.1177/0009922818769424 29644873

[B36] Hsu C. L. Lin J. C. C. (2016). Effect of perceived value and social influences on mobile app stickiness and in-app purchase intention. *Technol. Forecast. Soc. Change* 108 42–53. 10.1016/j.techfore.2016.04.012

[B37] Ifinedo P. (2016). Applying uses and gratifications theory and social influence processes to understand students’ pervasive adoption of social networking sites: perspectives from the Americas. *Int. J. Inf. Manage.* 36 192–206. 10.1016/j.ijinfomgt.2015.11.007

[B38] Irwin T. J. Riesel J. N. Ortiz R. Helliwell L. A. Lin S. J. Eberlin K. R. (2021). The impact of social media on plastic surgery residency applicants. *Ann. Plast. Surg.* 86 335–339. 10.1097/SAP.0000000000002375 32349083

[B39] Jackson D. L. Gillaspy J. A. Purc-Stephenson R. (2009). Reporting practices in confirmatory factor analysis: an overview and some recommendations. *Psychol. Methods* 14 6–23. 10.1037/a0014694 19271845

[B40] Jarman H. K. Marques M. D. McLean S. A. Slater A. Paxton S. J. (2021). Motivations for social media use: associations with social media engagement and body satisfaction and well-being among adolescents. *J. Youth Adolesc.* 10.1007/s10964-020-01390-z 33475925

[B41] Jin J. F. Ford M. T. Chen C. C. (2013). Asymmetric differences in work–family spillover in North America and China: results from two heterogeneous samples. *J. Bus. Ethics* 113 1–14. 10.1007/s10551-012-1289-3

[B42] Jin X. L. Yin M. J. Zhou Z. Y. Yu X. Y. (2021). The differential effects of trusting beliefs on social media users’ willingness to adopt and share health knowledge. *Inf. Process. Manage.* 58:102413. 10.1016/j.ipm.2020.102413

[B43] Jin X. L. Zhou Z. Y. Yu X. Y. (2019). Predicting users’ willingness to diffuse healthcare knowledge in social media a communicative ecology perspective? *Inf. Technol. People* 32 1044–1064. 10.1108/ITP-03-2018-0143

[B44] Jin Y. Li J. K. Geng R. l. (2021). Research on the Churn intention of health APP users from the perspective of privacy. *Modern Inf.* 41 67–77. 10.3969/j.issn.1008-0821.2021.01.008

[B45] Kehr F. Kowatsch T. Wentzel D. Fleisch E. (2015). Blissfully ignorant: the effects of general privacy concern, general institutional trust, and affect in the privacy calculus. *Inf. Syst. J.* 25 607–635. 10.1111/isj.12062

[B46] Kim B. Han I. (2009). What drives the adoption of mobile data services? An approach from a value perspective. *J. Inf. Technol.* 24 35–45. 10.1057/jit.2008.28

[B47] Kim C. Lee J. K. (2016). Social media type matters: investigating the relationship between motivation and online social network heterogeneity. *J. Broadcast. Electron. Media* 60 676–693. 10.1080/08838151.2016.1234481

[B48] Koufaris M. (2002). Applying the technology acceptance model and flow theory to online consumer behavior. *Inf. Syst. Res.* 13 205–223. 10.1287/isre.13.2.205.83 19642375

[B49] Lee H. Park H. Kim J. (2013). Why do people share their context information on social network services? A qualitative study and an experimental study on users’ behavior of balancing perceived benefit and risk. *Int. J. Hum. Comput. Stud.* 71 862–877. 10.1016/j.ijhcs.2013.01.005

[B50] Lee Z. W. Y. Cheung C. M. K. Chan T. K. H. (2014). Explaining the development of the excessive use of massively multiplayer online games: a positive-negative reinforcement perspective. *Hawaii Int. Conf. Syst. Sci.* 47 668–677. 10.1109/HICSS.2014.89

[B51] Li H. Y. Lu X. Y. Xie M. F. (2018). Research on privacy paradox in social network sites under the perspective of construal level theory. *J. China Soc. Sci. Tech. Inf.* 37 1–13. 10.3772/j.issn.1000-0135.2018.01.001

[B52] Li P. X. Cho H. C. Goh Z. H. (2019). Unpacking the process of privacy management and self-disclosure from the perspectives of regulatory focus and privacy calculus. *Telem. Inform.* 41 114–125. 10.1016/j.tele.2019.04.006

[B53] Li Q. Gao X. Y. (2019). The user sustained concern intension over corporate micro-blogs based on attachment theory. *J. Dalian Univ. Technol.* 40 7–14. 10.19525/j.issn1008-407x.2019.01.002

[B54] Lin H. F. (2008). Determinants of successful virtual communities: contributions from system characteristics and social factors. *Inf. Manage.* 45 522–527. 10.1016/j.im.2008.08.002

[B55] Lin H. Fan W. Chau P. Y. (2014). Determinants of users’ continuance of social networking sites: a self-regulation perspective. *Inf. Manage.* 51 595–603. 10.1016/j.im.2014.03.010

[B56] Mahmoodi J. Curdova J. Henking C. Kunz M. Matic K. Mohr P. (2018). Internet users’ valuation of enhanced data protection on social media: which aspects of privacy are worth the most? *Front. Psychol.* 9:1516. 10.3389/fpsyg.2018.01516 30186203 PMC6113717

[B57] Mikulincer M. Shaver P. R. (2007). Boosting attachment security to promote mental health, prosocial values, and intergroup tolerance. *Psychol. Inq.* 18 139–156. 10.1080/10478400701512646

[B58] Muhammad S. S. Dey B. L. Weerakkody V. (2018). Analysis of factors that influence customers’ willingness to leave big data digital footprints on social media: a systematic review of literature. *Inf. Syst. Front.* 20 559–576. 10.1007/s10796-017-9802-y

[B59] Mwencha P. M. Muathe S. M. Thuo J. K. (2014). Effect of perceived attributes, perceived risk and perceived value on usage of online retailing services. *J. Manage. Res.* 6 140–155.

[B60] Ohly S. Sonnentag S. Niessen C. Zapf D. (2010). Diary studies in organizational research: an introduction and some practical recommendations. *J. Pers. Psychol.* 9 79–93. 10.1027/1866-5888/a000009

[B61] Osatuyi B. (2013). Information sharing on social media sites. *Comput. Hum. Behav.* 29 2622–2631. 10.1016/j.chb.2013.07.001

[B62] Overby J. W. Lee E. J. (2006). The effects of utilitarian and hedonic online shopping value on consumer preference and intentions. *J. Bus. Res.* 59 1160–1166. 10.1016/j.jbusres.2006.03.008

[B63] Paolacci G. Chandler J. (2014). Inside the Turk: understanding mechanical turk as a participant pool. *Curr. Dir. Psychol. Sci.* 23 184–188. 10.1177/0963721414531598

[B64] Papacharissi Z. Rubin A. M. (2000). Predictors of internet use. *J. Broadcast. Electron. Media* 44 175–196. 10.1207/s15506878jobem4402_2

[B65] Peng L. H. Li H. Zhang Y. F. Hong C. (2018). Research on the influence factors of user privacy security on mobile social media fatigue behavior: based on privacy computing theory of CAC research paradigm. *Inf. Sci.* 36 96–102. 10.13833/j.issn.1007-7634.2018.09.017

[B66] Peng L. Xie T. (2016). Making similarity versus difference comparison affects perceptions after bicultural exposure and consumer reactions to culturally mixed products. *J. Cross Cult. Psychol.* 47 1380–1394. 10.1177/0022022116668409

[B67] Peters A. N. Winschiers-Theophilus H. Mennecke B. E. (2015). Cultural influences on Facebook practices: a comparative study of college students in Namibia and the United States. *Comput. Hum. Behav.* 49 259–271. 10.1016/j.chb.2015.02.065

[B68] Petrulaitiene V. Jylha T. (2015). The perceived value of workplace concepts for organisations. *J. Corp. Real Estate* 17 260–281. 10.1108/JCRE-06-2015-0014

[B69] Podsakoff P. M. MacKenzie S. B. Podsakoff N. P. (2012). Sources of method bias in social science research and recommendations on how to control it. *Annu. Rev. Psychol.* 63 539–569. 10.1146/annurev-psych-120710-100452 21838546

[B70] Punj G. N. (2019). Understanding individuals’ intentions to limit online personal information disclosures to protect their privacy: implications for organizations and public policy. *Inf. Technol. Manage.* 20 139–151. 10.1007/s10799-018-0295-2

[B71] Schomakers E. M. Lidynia C. Ziefle M. (2019). A typology of online privacy personalities exploring and segmenting users’ diverse privacy attitudes and behaviors. *J. Grid Comput.* 17 727–747. 10.1007/s10723-019-09500-3

[B72] Shin D. H. (2010). The effects of trust, security and privacy in social networking: a security-based approach to understand the pattern of adoption. *Interact. Comput.* 22 428–438. 10.1016/j.intcom.2010.05.001

[B73] Sit J. K. Birch D. (2014). Entertainment events in shopping malls-profiling passive and active participation behaviors. *J. Consum. Behav.* 13 383–392. 10.1002/cb.1487

[B74] Skoric M. M. Zhu Q. Goh D. Pang N. (2016). Social media and citizen engagement: a meta-analytic review. *New Media Soc.* 18 1817–1839. 10.1177/1461444815616221

[B75] Smith S. M. Roster C. A. Golden L. L. Albaum G. S. (2016). Amulti-group analysis of online survey respondent data quality: comparing a regular USA consumer panel to MTurk samples. *J. Bus. Res.* 69 3139–3148. 10.1016/j.jbusres.2015.12.002

[B76] Son J. Y. Kim S. S. (2008). Internet users’ information privacy-protective responses: a taxonomy and a nomological model. *MIS Q.* 32 503–529. 10.1128/AAC.02814-14 24957837 PMC4135866

[B77] Tan X. Qin L. Kim Y. Hsu J. (2012). Impact of privacy concern in social networking web sites. *Internet Res.* 22 211–233. 10.1108/10662241211214575

[B78] Tang X. J. Huang C. X. Wang D. (2019). “5G+AI” application scenario: personal data protection, new challenges and the responses. *Library* 12 43–49. 10.3969/j.issn.1002-1558.2019.12.002

[B79] Teo H. H. Chan H. C. Wei K. K. Zhang Z. (2003). Evaluating information accessibility and community adaptivity features for sustaining virtual learning communities. *Int. J. Hum. Comput. Stud.* 59 671–697. 10.1016/S1071-5819(03)00087-9

[B80] Tirukkovalluri S. S. Malarvannan K. Karthik R. C. Mahendiran B. S. Arumugam B. (2020). An observational exploration of factors affecting perceived social isolation among social media using medical professional course students in south Indian state of India. *Indian J. Commun. Health* 32 76–81.

[B81] Tsai T. H. Chang H. T. Chang Y. C. Chang Y. S. (2017). Personality disclosure on social network sites: an empirical examination of differences in Facebook usage behavior, profile contents and privacy settings. *Comput. Hum. Behav.* 76 469–482. 10.1016/j.chb.2017.08.003

[B82] Verbeke G. Fieuws S. Molenberghs G. (2014). The analysis of multivariate longitudinal data: a review. *Stat. Methods Med. Res.* 23 42–59. 10.1177/0962280212445834 22523185 PMC3404254

[B83] Walker G. J. (2020). Social psychology of leisure 2.0: looking back, looking forward. *J. Leis. Res.* 51 626–634. 10.1080/00222216.2020.1807845

[B84] Wang C. Zhou Z. Y. Jin X. L. Fang Y. L. (2017). The influence of affective cues on positive emotion in predicting instant information sharing on microblogs: gender as a moderator. *Inf. Process. Manage.* 53 721–734. 10.1016/j.ipm.2017.02.003

[B85] Wang L. Hu H. H. Yan J. Mei M. Q. Z. (2020). Privacy calculus or heuristic cues? The dual process of privacy decision making on Chinese social media. *J. Enterp. Inf. Manage.* 33 353–380. 10.1108/JEIM-05-2019-0121

[B86] Wang Y. W. (2020). Humor and camera view on mobile short-form video apps influence user experience and technology-adoption intent, an example of Tik Tok (DouYin). *Comput. Hum. Behav.* 110:106373. 10.1016/j.chb.2020.106373

[B87] Wang Y. Huang L. Y. (2017). Research on the impact of mobile short video perceived value on consumers’ purchase intention. *Econ. Manage.* 33 68–74.

[B88] Wu S. J. Bai X. C. Z. Fiske S. T. (2018). Admired rich or resented rich? how two cultures vary in envy. *J. Cross Cult. Psychol.* 49 1114–1143. 10.1177/0022022118774943

[B89] Xu S. Yang H. H. MacLeod J. Zhu S. (2019). Social media competence and digital citizenship among college students. *Converg. Int. J. Res. New Media Technol.* 25 735–752. 10.1177/1354856517751390

[B90] Xu Z. X. Ma H. K. (2016). How can a deontological decision lead to moral behavior? The moderating role of moral identity. *J. Bus. Ethics* 137 537–549. 10.1007/s10551-015-2576-6

[B91] Yang M. S. Hu S. G. Kpandika B. E. Liu L. (2021a). Effects of social attachment on social media continuous usage intention: the mediating role of affective commitment. *Hum. Syst. Manage.* 40 619–631. 10.3233/HSM-201057

[B92] Yang M. S. Zhang W. S. Ruangkanjanases A. Zhang Y. (2021b). Understanding the mechanism of social attachment role in social media: a qualitative analysis. *Front. Psycol.* 12:720880. 10.3389/fpsyg.2021.720880 34421773 PMC8378210

[B93] Yen Y. S. (2012). Exploring customer perceived value in mobile phone services. *Int. J. Mobile Commun.* 10 213–229. 10.1504/IJMC.2012.045674

[B94] Yin M. Li Q. (2017). Research on users’ continuance participation intention of microblog topics based on perceived value. *J. Intell.* 36 94–100. 10.3969/j.issn.1002-1965.2017.08.017

[B95] Yun H. J. Lee G. Kim D. J. (2019). A chronological review of empirical research on personal information privacy concern: an analysis of contexts and research constructs. *Inf. Manage.* 56 570–601. 10.1016/j.im.2018.10.001

[B96] Zhang C. B. Li Y. N. Wu B. Li D. J. (2017). How wechat can retain users: roles of network externalities, social interaction ties, and perceived values in building continuance intention. *Comput. Hum. Behav.* 69 284–293. 10.1016/j.chb.2016.11.069

[B97] Zhang M. F. Dawson J. F. Kline R. B. (2021). Evaluating the use of covariance-based structural equation modelling with reflective measurement in organizational and management research: a review and recommendations for best practice. *Br. J. Manage.* 32 257–272. 10.1111/1467-8551.12415

[B98] Zhao L. Lu Y. Wang B. Chau P. Y. K. Zhang L. (2012). Cultivating the sense of belonging and motivating user participation in virtual communities: a social capital perspective. *Int. J. Inf. Manage.* 32 574–588. 10.1016/j.ijinfomgt.2012.02.006

[B99] Zhao W. J. Zhou X. M. (2017). A research on the continuance intention of mobile SNS users from the view of perceived value. *Sci. Res. Manag.* 38 153–160. 10.19571/j.cnki.1000-2995.2017.08.018

